# Supplementary material for: Can Photoselective Nets’ Influence Pollen Traits? A Case Study in ‘Matua’ and ‘Tomuri’ Kiwifruit Cultivars
Source: Plants (Basel). 2024 Jun 19;13(12):1691. doi: 10.3390/plants13121691 (PMC11207864; doi:10.3390/plants13121691)
Supplement: Supplementary file 1 [file plants-13-01691-s001.zip › plants-2950477-supplementary.pdf]

Table S1 – Values of morphological and ultrastructure parameters of cv Matua and Tomuri pollen grains.

PNs / Matua	L (µm)	W (µm)	L/W	A (µm^2)	EM/ µm^2
Control	32.93±1.88	15.95±1.12	2.07±0.11	413.64±47.76	6.83±2.81
Yellow	32.92±1.56	16.38±1.19	2.02±0.14	423.95±42.65	6.36±4.20
Pearl	33.26±1.62	16.39±1.05	2.03±0.12	428.69±40.85	7.46±2.12
Grey	33.08±1.70	16.34±0.90	2.03±0.11	425.13±38.75	6.90±2.34
Tomuri					
Control	32.70±1.71	15.98±0.87	2.05±0.11	410.90±38.00	5.85±1.59
Yellow	33.32±1.69	16.14±1.01	2.07±0.12	423.01±40.86	8.21±3.37
Pearl	33.03±1.59	16.78±1.00	1.97±0.10	435.99±41.60	6.81±2.92
Grey	33.07±1.86	16.60±1.03	2.00±0.10	432.20±45.48	4.56±2.25

Values are presented as mean ± SD (n = 100). Length (L), width (W), L/W ratio, area (A), exine´s microperforations (EM). Cv: cultivar, PNs: photo-selective nets.

Photo-selective nets (PNs) colour			
Control	Yellow	Pearl	Grey

A. deliciosa cv. Matua

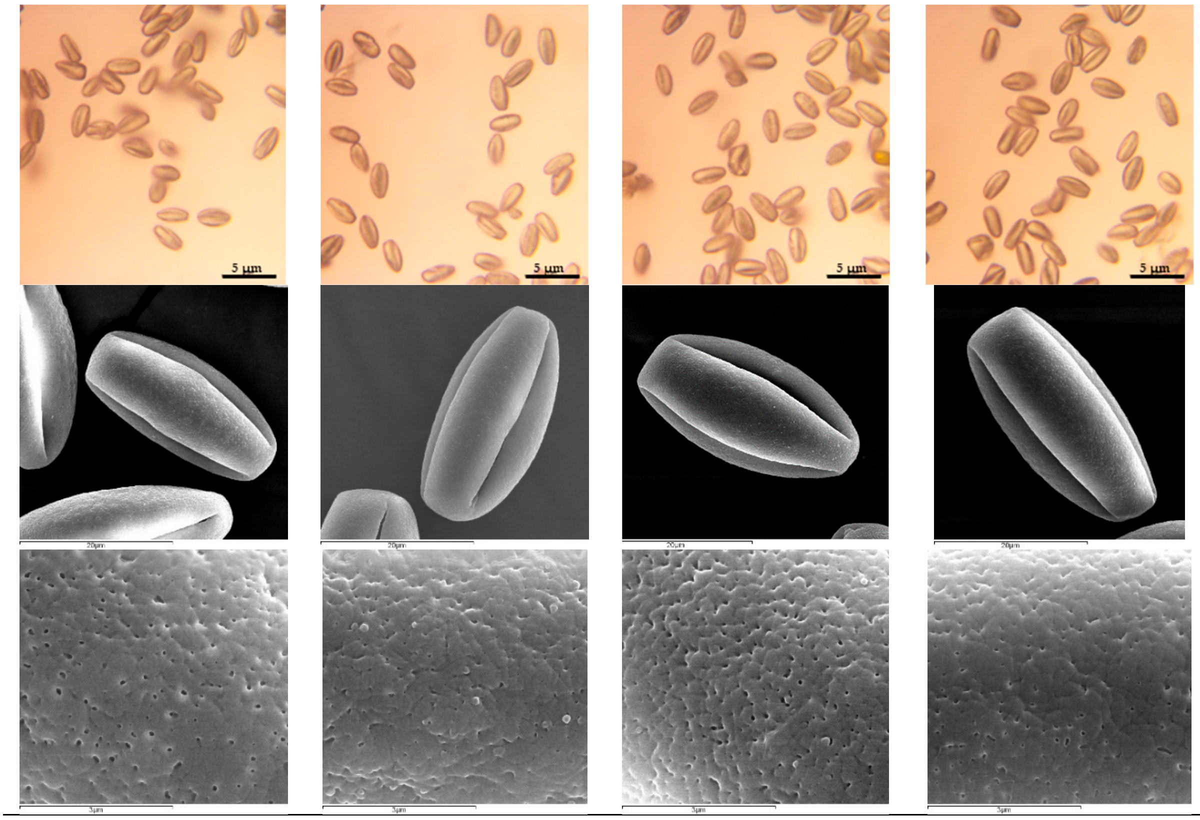

A. deliciosa cv. Tomuri

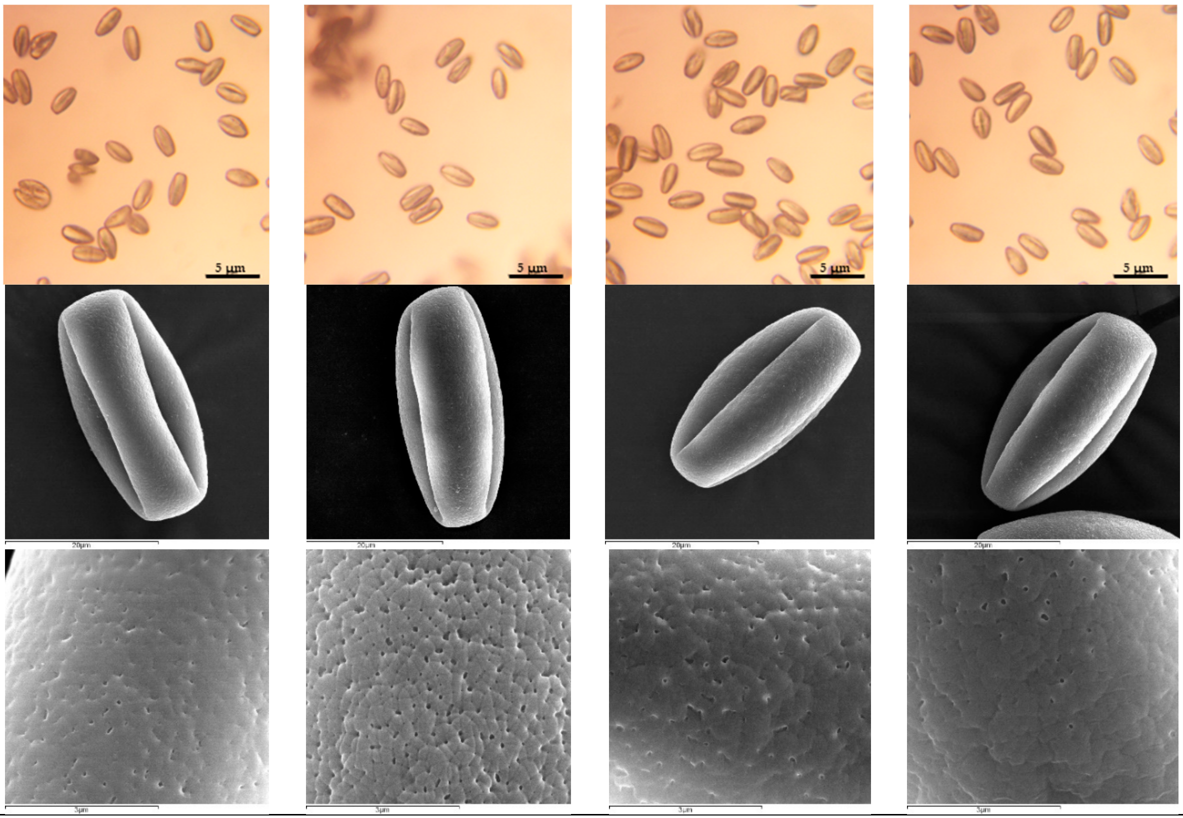

Figure S1 –Microphotograps of pollen (light microscopy and scanning electron microscopy-SEM) and exine’s microperfuration density (SEM) from *A. deliciosa* cv Matua and Tomuri grown under open field conditions (control) and yellow, pearl and grey net.

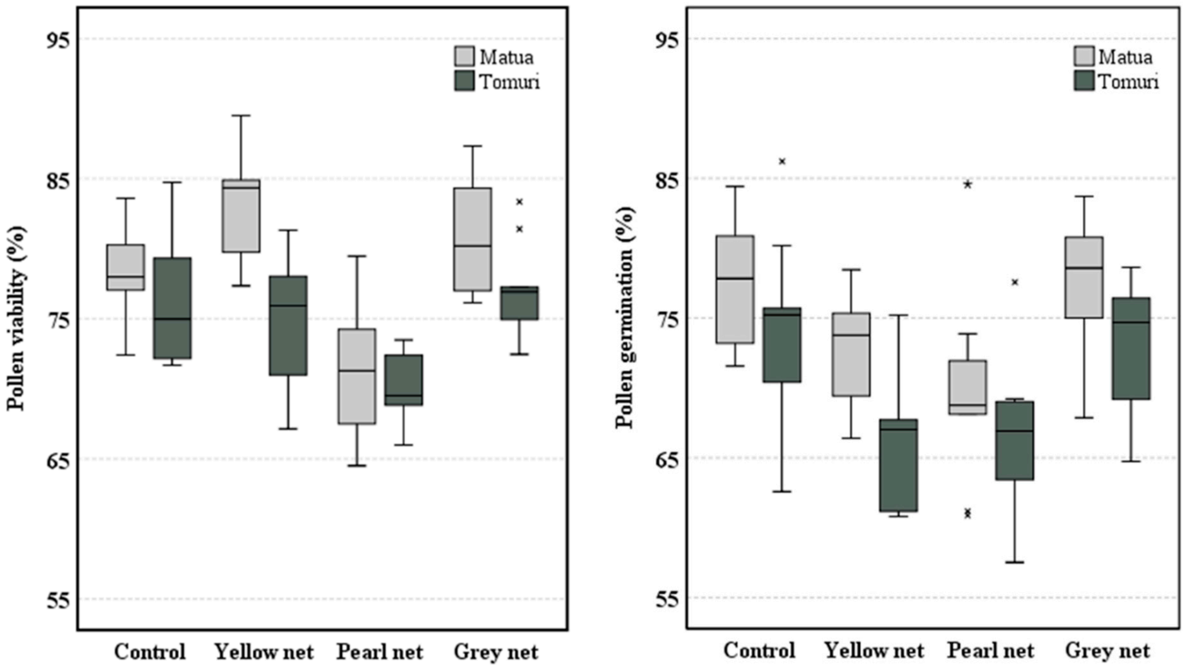

Figure S2 – Box-plots of pollen viability and germination from cv Matua and Tomuri (quartiles with the median and outliers indicated by stars).

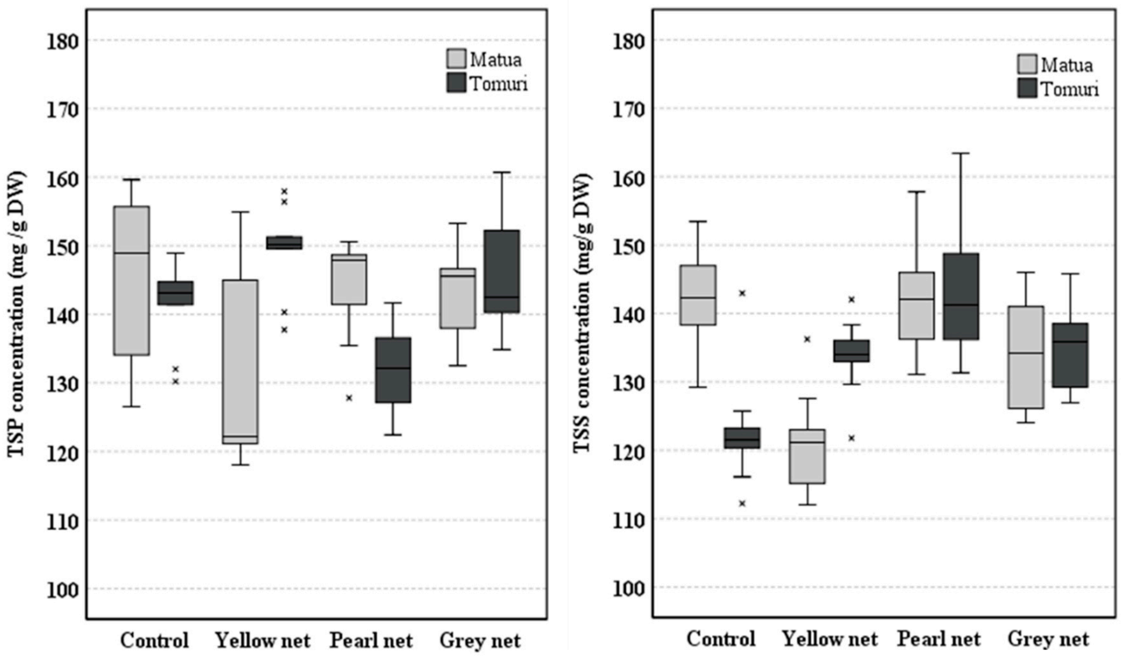

Figure S3 – Box-plots of total soluble proteins (TSP) and total soluble sugars (TSS) of pollen from cv Matua and Tomuri (quartiles with the median and outliers indicated by stars).

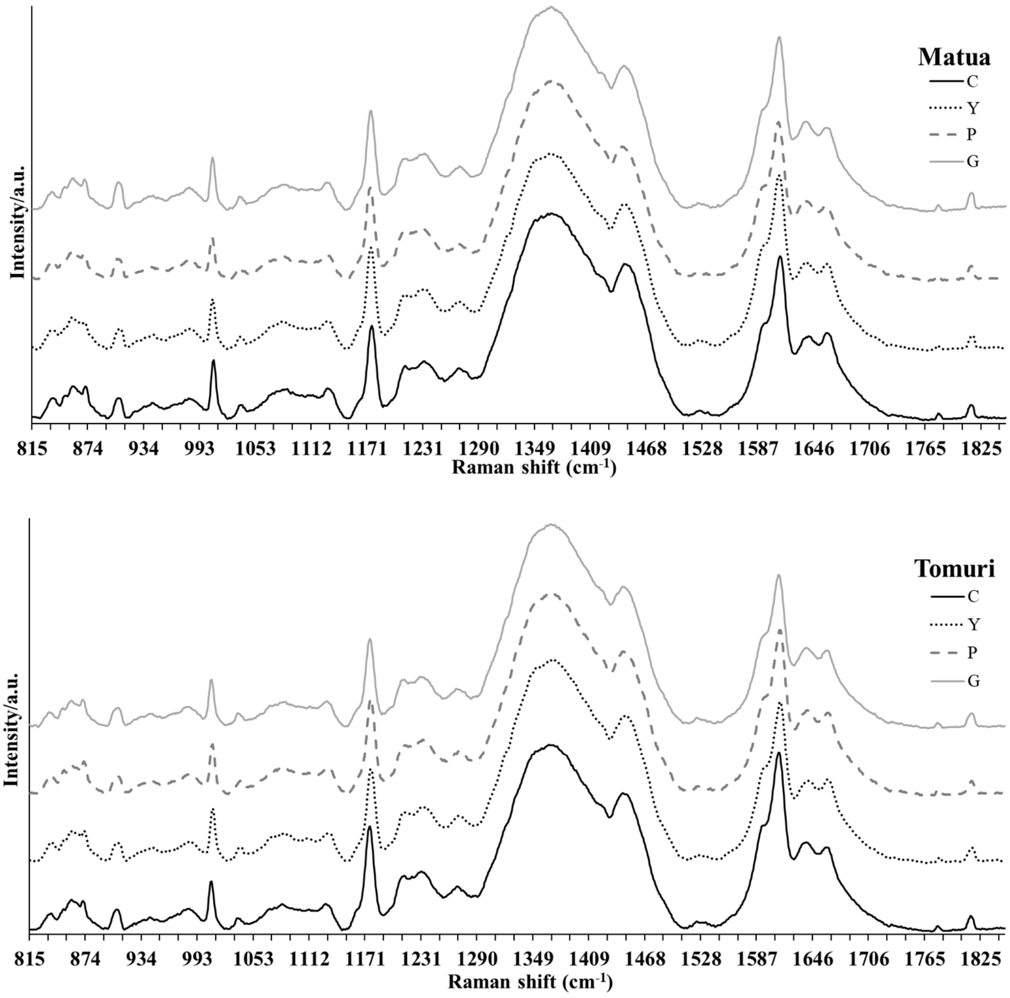

Figure S4 – Raman spectra of cv Matua and Tomuri pollen.

Table S2 – Wavenumber interval of the Raman spectra peak calculated by deconvolution of the spectra of Matua and Tomuri pollen grains. Results of one-way ANOVA test followed by Duncan *pos-hoc* test ($p < 0.01$) for pairwise comparisons with subsets formed indicating groups with significantly different mean values.

Cv	Wavenumber cm^{-1}	ANOVA Sig.	Subsets of PNs after Duncan's pos-hoc test n = 10; α = 0.01			
Matua	1006-1008	0.000	Control 1008	Yellow 1007	Grey 1007	Pearl 1006.5
	1127-1131	0.000	Control 1130.4	Grey 1129.1	Yellow 1128.8	Pearl 1128.5
	1174-1176	0.000	Control 1176	Yellow 1175	Grey 1175	Pearl 1174.3
	1207-1210	0.000	Control 1209.5	Yellow 1208.5	Grey 1208.4	Pearl 1207.9
	1609-1611	0.000	Control 1610.8	Grey 1610	Yellow 1609.9	Pearl 1609.1
Tomuri	1007-1010	0.000	Control 1008.1	Grey 1008	Pearl 1009	Yellow 1009.2
	1036-1039	0.000	Control 1036.9	Grey 1036.9	Pearl 1038.1	Yellow 1038.1
	1128-1133	0.000	Control 1029.7	Grey 1130.1	Pearl 1131.7	Yellow 1131.8
	1175-1177	0.000	Control 1175.8	Grey 1175.8	Pearl 1177	Yellow 1177
	1208-1211	0.000	Control 1209.1	Grey 1209.3	Pearl 1210.3	Yellow 1210.8
	1610-1612	0.000	Control 1610.2	Grey 1610.5	Pearl 1611.5	Yellow 1611.9

Cv: cultivar, PNs: photo-selective nets, sig: significance.
